# The Role of Immunological Bystander Reaction in Recurrent Pregnancy Loss

**DOI:** 10.7759/cureus.106246

**Published:** 2026-03-31

**Authors:** Igor Lakhno

**Affiliations:** 1 Obstetrics and Gynecology, Kharkiv National Medical University, Kharkiv, UKR

**Keywords:** chronic inflammation, immune therapy in pregnancy, immunological bystander reaction, recurrent pregnancy loss, thrombophilia

## Abstract

Recurrent pregnancy loss (RPL) affects approximately 1%-2% of women of reproductive age and may be associated with immunological bystander reactions. These reactions occur when the maternal immune system's response to a specific target extends to nearby tissues, causing collateral damage to both maternal and fetal cells. This narrative review explores the mechanisms of immune dysregulation at the maternal-fetal interface and evaluates current therapeutic strategies.

A literature search for a narrative review was conducted using PubMed, Scopus, and Web of Science for the period 2010-2025. Keywords included "immunological bystander reaction," "recurrent pregnancy loss," and "immune therapy in pregnancy". Following a Preferred Reporting Items for Systematic Reviews and Meta-Analyses (PRISMA)-based screening process, 41 reports were selected for final analysis.

A successful pregnancy requires the maternal immune system to maintain a delicate balance between protecting against pathogens and tolerating the semi-allogeneic fetus. Bystander activation, often mediated by soluble cytokines, allows non-antigen-specific T cells to proliferate and cause tissue damage without prior sensitization. A critical factor is the Th1/Th2 cytokine balance: pro-inflammatory Th1 cytokines (e.g., tumor necrosis factor alpha (TNF-α), interferon gamma (IFN-γ)) are associated with fetal resorption, while Th2 cytokines (e.g., interleukin (IL)-4, IL-10) promote immune tolerance. The review highlights a significant shift in clinical understanding, particularly the work of Professor Hassan Shehata, who challenges the traditional focus on thrombophilia. Data shows that the prevalence of inherited thrombophilia in RPL patients (8.1%) is nearly identical to the general population, suggesting that "sticky blood" is not the primary driver in most cases. Instead, the focus is moving toward reproductive immunology, specifically the role of overactive natural killer (NK) cells and cytokine imbalances.

According to the 2025 American Society for Reproductive Immunology (ASRI) guidelines, management is shifting toward precision medicine. Prednisolone is recommended for women with documented immune abnormalities (e.g., uterine NK (uNK) cells >5% or antinuclear antibody (ANA) positive) to suppress inflammation and promote Th2 dominance. Hydroxychloroquine (HCQ) is conditionally recommended for established autoimmune diseases or specific inflammatory placental pathologies. Tacrolimus, a granulocyte-colony stimulating factor (G-CSF), and intralipids are currently categorized as having unclear benefits due to a lack of robust randomized controlled trial evidence.

Maternal inflammation is a primary trigger for disturbed placentation and fetal loss. Improving outcomes for women with RPL requires a proactive, evidence-based approach that specifically targets the immunological bystander reaction pathway rather than relying on routine clotting tests.

## Introduction and background

Recurrent pregnancy loss (RPL) is a significant reproductive health issue characterized by the repeated failure to maintain a pregnancy, affecting an estimated 1%-2% of women of reproductive age [1, 2]. A growing body of research suggests that immunological factors, particularly bystander immunological reactions, play a crucial role in the etiology of RPL. These reactions occur when the immune system mounts responses that extend beyond the specific antigens initially targeted, potentially leading to collateral damage to both maternal and fetal tissues. This phenomenon underscores the delicate balance the maternal immune system must maintain to tolerate the genetically distinct fetus while protecting against pathogens and ensuring a successful pregnancy [3,4]. The complex interplay between maternal immune adaptations and bystander activation of immune cells can contribute to inflammation and tissue damage, both of which are implicated in recurrent pregnancy loss [5,6]. Current clinical strategies are shifting away from routine thrombophilia testing toward precision medicine and immunotherapies such as prednisolone, although their use remains controversial in major international guidelines due to varying levels of robust clinical evidence [2,6]. Various immune cells, including natural killer (NK) cells, macrophages, and T cells, are pivotal in mediating these responses. Dysregulation of these immune mechanisms may result in excessive inflammation or inadequate immune tolerance, both of which can adversely affect pregnancy outcomes [7,8]. Controversies surrounding RPL often focus on the varying contributions of genetic, environmental, and immunological factors, complicating the understanding and management of the condition [9,10]. Bystander immunological reactions may amplify the risk of RPL through mechanisms such as cytokine-mediated activation of non-specific immune cells, which can lead to unintended consequences, including epitope spreading and autoimmune conditions [11,12]. This understanding has prompted researchers to explore therapeutic interventions that address these immunological imbalances, intending to improve pregnancy outcomes for affected individuals [13,14]. As such, the study of bystander reactions in the context of RPL represents a promising area of research with significant implications for clinical practice and patient care.

Literature search

**Figure 1:**
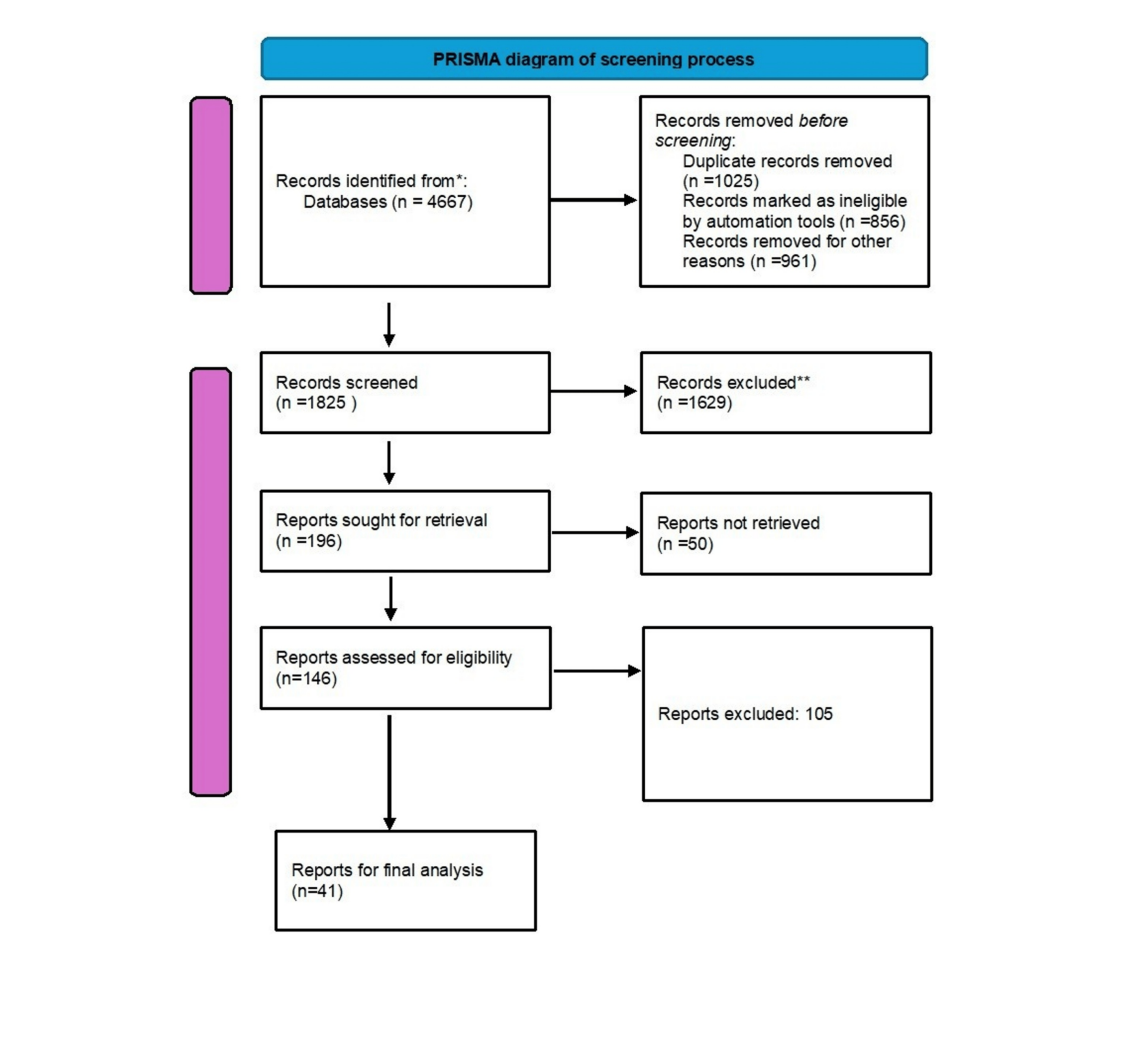
The stages of paper selection included in the study Preferred Reporting Items for Systematic Reviews and Meta-Analyses (PRISMA) flowchart.

For this narrative review, literature searches were performed using PubMed, Scopus, or Web of Science from 2010 to 2025. The following keywords were used: “immunological bystander reaction”, “recurrent pregnancy loss”, or “immune therapy in pregnancy”. The inclusion criteria were three or more consecutive or non-consecutive miscarriages; published in English; and randomized controlled trial comparing any types of diagnosis and treatment. The exclusion criteria were threatened miscarriage without a prior spontaneous abortion. The articles included in this review were selected based on their relevance to the topic. The results were further screened according to the title and abstract and whether they included animal experiments, in vitro studies, clinical trials, and database or software applications. After this search, 41 studies were finally considered for the review (see Figure 1).

## Review

Immunological mechanisms in pregnancy

Pregnancy represents a unique immunological paradigm where the maternal immune system must adapt to protect both the mother and the developing fetus from various pathogens while simultaneously tolerating the semi-allogeneic fetal tissues [1,2]. This delicate balance is critical for successful implantation, fetal development, and the overall maintenance of pregnancy.

Maternal immune adaptations

During pregnancy, the maternal immune system undergoes significant adaptations that are essential for healthy fetal development. These adaptations involve a complex interplay between maternal immune cells and trophoblastic cells from the fetus, facilitating proper implantation and protecting against infections [2,3]. Key immune cells at the maternal-fetal interface include decidual natural killer (dNK) cells, macrophages, and T cells, which play vital roles in mediating maternal tolerance and regulating immune responses [4].

Role of cytokines

Cytokines, produced by various immune cells, are crucial for orchestrating the immune responses during pregnancy. Maternal immune stimulation leads to elevated levels of pro-inflammatory cytokines in the serum and amniotic fluids, which can influence the pregnancy outcome [4,5]. Aberrant production of these cytokines can contribute to complications such as recurrent spontaneous miscarriage, pre-eclampsia, and pre-term labor [6].

The maternal-fetal interface

The maternal-fetal interface is a highly specialized environment where maternal immune cells interact closely with fetal-derived trophoblasts. This interface consists of multiple cellular layers, including the syncytiotrophoblast and decidual cells, which are essential for maintaining local immune homeostasis while providing a barrier against pathogen invasion [7,8]. Proper communication and signaling between maternal immune cells and fetal cells are critical to achieving a successful pregnancy outcome [2,9].

Implications of immune dysregulation

Dysfunctional interactions at the maternal-fetal interface can lead to significant pregnancy complications. For instance, abnormalities in the immune response can result in implantation failure or miscarriage due to an inadequate immune tolerance to the paternal antigens present in the fetus [2,6]. The recognition of this complex interplay highlights the importance of the immune system's adaptation during pregnancy and its implications for reproductive success.

Bystander immunological reactions

Bystander immunological reactions are critical phenomena where immune responses extend beyond the specific antigens initially targeted, affecting nearby cells that were not directly involved in the initial immune activation. This activation is often mediated by soluble factors such as cytokines, which can influence non-specific immune cells, leading to broader immunological effects [10].

Mechanisms of Bystander Activation

Bystander activation refers to the process by which immune cells, particularly T cells, respond to antigens without prior specific sensitization. This can occur when infected cells produce inflammatory signals that activate neighboring uninfected cells to mount an immune response. For instance, cytokines released during an immune response can trigger non-antigen-specific T cells to proliferate and become activated, a process termed "bystander T cell activation" [11]. This phenomenon underscores the interconnectedness of immune responses, where even T cells that do not recognize the primary pathogen can become activated due to the inflammatory environment established by the immune system [8,12].

Implications in Pregnancy

In the context of pregnancy, the bystander effect can manifest significantly, particularly in relation to recurrent pregnancy loss. The maternal immune system undergoes considerable changes to tolerate the genetically distinct fetus, which can inadvertently lead to bystander activation of immune cells that may cause harm [13,14]. Such misdirected immune responses can lead to inflammation and tissue damage, posing risks to both the mother and the developing fetus [7]. The heightened susceptibility to infections during pregnancy may also exacerbate bystander effects, further complicating immune balance [15].

Consequences of Bystander Activation

While bystander activation can enhance immune surveillance, it may also lead to collateral damage. Excessive immune activation may result in epitope spreading, where damaged cells release new antigens, potentially leading to autoimmune conditions [16,17]. The intricate balance between effective immune responses and the risks of bystander effects is crucial in maintaining both maternal health and fetal development [13,14]. As such, understanding these mechanisms is vital for developing therapeutic strategies to manage complications associated with pregnancy, including recurrent pregnancy loss.

Connection between bystander reactions and recurrent pregnancy loss

Bystander immunological reactions have emerged as a significant area of research in understanding RPL. The etiologies of RPL are complex and multifaceted, with only a few causes universally recognized, such as parental chromosomal abnormalities and thrombotic complications related to antiphospholipid syndrome (APS). Most studies identify additional factors that may contribute to RPL, including uterine anatomic abnormalities, endocrine issues, infections, and various immunologic factors, including those associated with APS [18,19]. Recent investigations into reproductive immunology suggest that alterations in immune responses could play a pivotal role in both isolated and recurrent cases of pregnancy loss. Many of these studies have focused on how distinct immune alterations might contribute to pregnancy outcomes. The interactions between immune factors and other variables, such as genetic and environmental influences, complicate the understanding of RPL [18,20,21]. Importantly, it has been noted that the immune responses during early pregnancy may be affected by the presence of bystander reactions, which can amplify the risk of pregnancy loss [22]. Research indicates that a range of immune dysfunctions may manifest in women with RPL, underscoring the need for more comprehensive immune assessments in affected populations [21,22]. The clinical study of immunological factors in RPL is further complicated by the fact that many patients seek evaluation only after experiencing pregnancy loss, potentially masking underlying immunologic issues that could be present even during successful pregnancies [18,21]. Moreover, the psychological impact of RPL cannot be overlooked, as many patients experience heightened emotional responses, which may be exacerbated by the uncertainty surrounding the causes of their losses. Psychological factors may intertwine with immune responses, creating a complex interplay that further complicates the management of RPL cases [21,23]. Understanding the implications of bystander immunological reactions thus holds promise for developing targeted therapies and enhancing patient care strategies in managing recurrent pregnancy loss. Such immunological disorders could be involved in chronic inflammation and secondary pathogenic events within the mother-placenta-fetus system. 

Overview of bystander reactions

Bystander reactions during pregnancy refer to immune responses that, although intended for the mother, inadvertently affect the fetus, which is considered a bystander in this context [12]. These reactions can result in various complications, including RPL and other adverse pregnancy outcomes [4].

Maternal Immune Response

The maternal immune system plays a crucial role in maintaining pregnancy. However, dysregulation of this immune response can lead to conditions such as pre-eclampsia, recurrent spontaneous miscarriage, and pre-term labor [4, 24]. Maternal immune cells, including lymphocytes, macrophages, and natural killer (NK) cells, have been extensively studied for their roles in these complications [24]. The cytokines produced by these immune cells are particularly significant in influencing the overall maternal-fetal immune environment.

Immune Cell Types and Functions

Innate immune cells, especially uterine NK (uNK) cells, macrophages, and dendritic cells, are abundant at the maternal-fetal interface. These cells not only participate in the initial immune response but also play essential roles in placental development and the establishment of immune tolerance [25]. This tolerance is crucial for preventing the maternal immune system from attacking the developing fetus, which carries paternal antigens that could be recognized as foreign [4].

Genetic Factors and Immune Tolerance

Genetic factors may also influence bystander reactions during pregnancy. Structural genetic abnormalities in one or both parents can lead to an increased risk of pregnancy loss [21]. Genetic counseling and preimplantation genetic testing can help identify at-risk couples and inform them of potential treatment options to improve outcomes [21].

Environmental and Hormonal Influences

Environmental factors and hormonal changes during pregnancy further modulate immune responses. For instance, hormonal fluctuations can affect immune marker expression, thereby influencing the balance between pro-inflammatory and anti-inflammatory signals at the maternal-fetal interface [24]. These changes can either support pregnancy by promoting tolerance or contribute to adverse outcomes if the balance is disrupted.

Interactions Between Immune Mediators

Interactions among immune mediators play a crucial role in the dynamics of immune responses, particularly in RPL. The immune system is equipped with various cells and molecules that protect against pathogens, but these same mechanisms can contribute to adverse outcomes such as RPL through inappropriate immune reactions [4].

Cytokine Activation and Bystander Responses

Cytokines are key signaling molecules that facilitate communication between immune cells. They can activate a broad range of immune cells, including T cells, even those that are not specifically targeting the original non-self antigen. This phenomenon, known as “bystander T cell activation,” occurs when antigen non-specific T cells are activated by cytokines present during an immune response, potentially leading to collateral damage to host cells and subsequent autoimmunity [8,26]. Bystander activation is particularly pronounced in memory T cells, which are more sensitive to cytokines than naive T cells. This heightened sensitivity allows for a more rapid immune response but can also result in increased tissue damage, underscoring the delicate balance between protective immunity and pathological inflammation [26]. In the context of RPL, inflammatory environments - often triggered by infections or tissue injury - can lead to the release of cytokines that activate T cells in an antigen-independent manner. The activated T cells then produce effector cytokines and chemokines that can recruit additional immune cells, further exacerbating inflammation in the affected tissues [27].

Role of Th1 and Th2 Cytokines

The immune response is characterized by the interplay between different subsets of T helper cells, primarily Th1 and Th2. Th1 cytokines are typically pro-inflammatory and can negatively affect pregnancy outcomes, as evidenced by studies showing that the administration of Th1 cytokines like tumor necrosis factor alpha (TNF-α) and interferon gamma (IFN-γ) in pregnant mice leads to fetal resorption [11]. In contrast, Th2 cytokines, such as interleukin (IL)-4 and IL-10, are associated with humoral immunity and can promote the production of antibodies by B cells, thereby playing a protective role during pregnancy [4]. The mutual inhibition between Th1 and Th2 cells further complicates the immune landscape, as imbalances in this interaction can lead to undesirable immune responses that jeopardize pregnancy viability [11].

Hormonal Modulation of Immune Interactions

Hormonal factors, particularly estrogen and progesterone, significantly influence immune responses during pregnancy. These hormones have immunomodulating effects that can alter cytokine production and immune cell activity, thereby shaping the maternal immune environment. For instance, progesterone has been shown to inhibit the proliferation of CD8+ T cells and alter cytokine responses, potentially fostering a microenvironment that supports pregnancy maintenance [19]. However, the immunosuppressive state induced by these hormones may increase susceptibility to infections, complicating the overall immune response during pregnancy [14].

Implications for treatment and management

The management of RPL has increasingly focused on the role of immunological factors, particularly regarding the modulation of cytokine responses. Current therapeutic strategies emphasize the necessity for a comprehensive approach that restores the balance of pro-inflammatory and anti-inflammatory cytokines, rather than targeting individual cytokines in isolation. For example, agents like lactoferrin and glucocorticoids have shown promise in managing inflammatory responses associated with RPL [28].

Early Intervention

It is critical to initiate treatment at the earliest onset of symptoms to maximize effectiveness. Delayed intervention may diminish the potential benefits, especially in cases where inflammation and thrombosis are already advanced [28]. Therefore, a proactive approach that combines immunomodulatory therapies, such as tocilizumab, may improve outcomes for patients experiencing severe cases of RPL [19,28].

Immunostimulating Therapies

Patients often seek therapies that leverage well-known cardiovascular benefits, such as low-dose aspirin (ASA). However, evidence supporting the efficacy of ASA in preventing RPL, particularly among those with unexplained cases, remains limited [18, 19]. In cases of antiphospholipid syndrome (APS), the combination of low-dose ASA with unfractionated heparin has demonstrated superior live-birth rates compared to ASA alone, highlighting the need for tailored treatment protocols in immunological contexts [21].

Controversies in RPL 

Professor Hassan Shehata has dwelt on significant controversies regarding the routine screening and treatment of thrombophilia in women with RPL, arguing that the medical community currently overdiagnoses and overtreats these conditions without improving clinical outcomes. The core controversy is around the question: Is thrombophilia actually higher in RPL? Professor Shehata challenges the widely held belief that inherited or acquired blood-clotting disorders are a primary cause of early miscarriage. His research aims to correct what he describes as "disproportionally high" prevalence reports in previous literature [29-31]. In a large cohort study of 1,155 women with three or more first-trimester miscarriages, Shehata found the prevalence of inherited thrombophilia (such as Factor V Leiden or prothrombin gene mutations) to be 8.1%. Using strict diagnostic criteria (the revised Sapporo criteria), Shehata found that true antiphospholipid syndrome (APS) is extremely rare, occurring in only 1% of his RPL cohort (or 0.83% according to another of his studies). 

Recommendation to stop testing

Because the prevalence of these clotting factors in women with RPL does not exceed that of the general population, Professor Shehata's clinical position is controversial but firm: he does not recommend the routine investigation or treatment of inherited or acquired thrombophilia for women with early recurrent miscarriage. He suggests that testing for "sticky blood" has become a "classic" investigation that only explains a tiny minority of cases [29].

Shifting focus: from clotting to immunology

The central controversy in Shehata’s approach is the shift away from hematology (clotting) toward reproductive immunology. He argues that "classic" investigations often fail to identify a reason for loss, leaving many cases "unexplained" when the actual cause may be an overactive immune system.

The Role of NK Cells

Instead of focusing on microthrombi (clots) in the placenta, Shehata emphasizes the role of NK cells (specifically CD16+, CD56+, and CD69+), which he believes can attack the uterus or prevent embryo attachment.

Cytokine Imbalance

He highlights that an imbalance in Th1 (pro-inflammatory) and Th2 (anti-inflammatory) cytokines is a more likely driver of pregnancy loss than clotting issues for the majority of patients.

Treatment Disparity

While conventional protocols often rely on heparin and aspirin "just in case," Shehata advocates for targeted immunotherapy, such as prednisolone, hydroxychloroquine, or intralipids, only when specific immune biomarkers are confirmed.

In summary, Shehata's work suggests that the medical community's obsession with thrombophilia in early RPL is largely misplaced. He contends that doctors should stop routine clotting tests and instead investigate the complex immunological environment of the mother, which he believes provides the real answers for most "unexplained" losses.

Novel approaches

Research is shifting towards innovative immunomodulatory strategies that fine-tune immune responses at the maternal-fetal interface. These approaches aim to selectively enhance protective immune responses while minimizing harmful inflammation. For instance, targeting specific signaling pathways and utilizing small-molecule inhibitors that block detrimental cytokines may provide new avenues for treating RPL [7,21,32,33].

According to the 2025 American Society for Reproductive Immunology (ASRI) guidelines and other contemporary research, the use of these therapies for RPL is increasingly tied to specific immune biomarkers, though many treatments remain controversial within the wider medical community [34-36]. The summary of these interventions is presented in Table 1.

**Table 1:** Summary of treatment status The evidence-based effects of interventions. ASRI: American Society for Reproductive Immunology; ANA: anti-nulear antibody; uNK: uterine natural killer cells; RCT: randomized controlled trial; G-CSF: granulocyte colony stimulating factor.

Medication	ASRI 2025 Recommendation	Evidence Level / Notes
Prednisolone	Recommended	Strongest evidence for ANA+ or uNK >5%
Hydroxychloroquine	Conditionally Recommended	Best for autoimmune or placental inflammation
Tacrolimus	Unclear Benefit	Requires more research on Th1/Th2 ratios
G-CSF	Unclear Benefit	Conflicting trial results; possible side effects
Intralipid	Unclear Benefit	Experimental; lacks strong RCT data

Prednisolone (corticosteroids)

Prednisolone is an oral anti-inflammatory used to suppress immune responses and shift the maternal environment toward Th2 dominance.

ASRI Recommendations

It is strongly recommended for women with unexplained RPL who have documented immune abnormalities, such as being antinuclear antibody (ANA) positive, having uterine NK (uNK) cells >5%, or other positive autoantibodies. It is conditionally recommended for those without documented abnormalities, as it may address undetected immune issues.

Clinical Data

Meta-analyses show increased live birth rates in specific subgroups, such as those with elevated uNK cells.

Controversy

Organizations like the European Society of Human Reproduction and Embryology (ESHRE) and American Society for Reproductive Medicine (ASRM) do not recommend corticosteroids due to a lack of high-quality, large-scale evidence.

Safety

While generally safe, long-term use slightly increases the risk of preterm birth, low birth weight, and preeclampsia.

Hydroxychloroquine (HCQ)

HCQ, an oral medication originally for malaria, is now utilized for its immunomodulatory effects in autoimmune pregnancy complications.

ASRI Recommendation

It is conditionally recommended for women with established autoimmune diseases (like lupus) or specific inflammatory placental pathologies, such as chronic intervillositis.

Dosing

A typical regimen is 100-200 mg twice daily, continued throughout pregnancy and often postpartum.

Safety

HCQ is considered generally safe during pregnancy, with rare side effects related to the eyes or muscles.

Tacrolimus (calcineurin inhibitor)

Tacrolimus is an oral immunosuppressant used off-label to modulate Th1/Th2 ratios and NK cell activity.

ASRI Status

The guidelines state that the benefit is currently unclear. While small studies show improved outcomes in women with Th1/Th2 imbalances, more research is required to standardize its use.

Safety

It is considered safe at low doses, but monitoring for kidney strain and high blood sugar is necessary.

Granulocyte-Colony Stimulating Factor (G-CSF)

G-CSF is an injectable medication that stimulates the immune system and is theorized to improve endometrial receptivity and promote Th2 responses.

ASRI Status

The benefit is listed as unclear. Clinical trials have yielded conflicting results depending on the timing and route of administration, and further randomized controlled trials are needed.

Side Effects

Common mild reactions include fatigue and bone pain.

Intralipid (intravenous lipid emulsion)

Intralipid is an IV nutritional supplement made of fats (soy or egg-based) used off-label to suppress NK cell activity and inhibit pro-inflammatory cytokines.

ASRI Status

The benefit is currently unclear due to a lack of robust randomized controlled trial (RCT) evidence.

Safety

It should be strictly avoided in patients with allergies to soy, egg, or peanut, or those with fat metabolism disorders. The sources emphasize that these therapies represent a shift toward precision medicine, where treatment should ideally be based on documented immune dysfunction rather than being applied broadly to all RPL patients.

The holistic approach postulates the current strategy in immune-mediated RPL [37-39]. Maternal inflammation is a trigger for the disturbed placentation and fetal loss [40,41]. Multiple periconceptional interventions contribute to improved outcomes in RPL.

Analysis of key included studies and guidelines

Table 2 summarizes the primary studies and consensus guidelines that form the basis of the review's conclusions.

**Table 2:** Recurrent Pregnancy Loss (RPL) and immunological bystander reactions The analysis of key included studies and guidelines. HCQ: Hydroxychloroquine; G-CSF: granulocyte colony stimulating factor; ANA: antinuclear antibody; uNK: uterine natural killer cells; RCT: randomized controlled trial; APS: antiphospholipid syndrome

Study / Guideline	Year	Type	Sample Size/Focus	Intervention/Exposure	Key Results/Effect Estimates
Shehata et al. [29]	2022	Large Cohort Study	1,155 women (3+ miscarriages)	Inherited and acquired thrombophilia screening	8.1% prevalence of inherited thrombophilia (identical to the general population); 1% prevalence of APS. Recommended stopping routine testing.
ASRI Guidelines [34]	2025	Practice Recommendations	Immunological interventions in RPL	Prednisolone, HCQ, Tacrolimus, G-CSF, Intralipids	Prednisolone is strongly recommended for ANA+ or uNK >5% patients; others have "unclear" benefits due to lack of RCTs.
Rasmark Roepke et al. [35]	2019	Randomized Controlled Trial (RCT)	Unexplained RPL	Low-molecular-weight heparin (LMWH)	LMWH increases Th1- and Th17-associated chemokine levels, potentially complicating unexplained cases.
Moustakli et al. [18]	2025	Systematic Review	Immunological factors in RPL	Cytokine pathways and emerging therapies	Identifies bystander reactions as an amplifier of pregnancy loss risk through non-specific immune activation.

Risk of bias assessment

The sources evaluate the scientific merit of current RPL treatments based on the strength of clinical evidence and the presence of conflicting trial data. A generalized risk of bias assessment based on the "evidence levels" provided in the sources is as follows.

Selection Bias

The review notes that many patients only seek evaluation after loss, which may mask underlying issues present in successful pregnancies, introducing a potential selection bias in clinical studies.

Reporting/Publication Bias

There is a noted lack of high-quality, large-scale evidence for many immunomodulatory treatments, such as corticosteroids, leading to organizations like ESHRE and ASRM withholding recommendations despite positive, smaller meta-analyses.

Conflict of Evidence

Treatments like G-CSF and intralipids are currently categorized as having "unclear benefit" because existing clinical trials have yielded conflicting results or lack robust RCT data.

Methodological Rigor

The shift toward precision medicine aims to reduce bias by targeting treatment only to those with confirmed immune biomarkers (e.g., uNK cells >5%), rather than applying broad therapies to unexplained cases.

## Conclusions

In summary, the traditional medical approach to RPL, which heavily favored screening for blood-clotting disorders (thrombophilia), is undergoing a significant paradigm shift. Evidence now suggests that inherited clotting factors are no more common in women with RPL than in the general population, making routine "sticky blood" testing an outdated practice for the vast majority of patients. Instead, the focus has moved to the maternal-fetal interface and the complex immunological bystander reactions that occur there.

A successful pregnancy requires a "Goldilocks" immune state - enough activity to protect against infection but enough tolerance to protect the semi-allogeneic fetus. When this balance is disrupted, often by maternal inflammation or infection, the immune system can inadvertently attack fetal tissues. This "bystander" damage, driven by an imbalance of pro-inflammatory (Th1) and anti-inflammatory (Th2) cytokines, appears to be a pivotal factor of previously "unexplained" losses.

The future of RPL management lies in precision medicine. Rather than a one-size-fits-all approach using aspirin or heparin "just in case," modern strategies emphasize the use of targeted immunotherapies. By identifying specific biomarkers, such as elevated NK cells or specific autoantibodies, clinicians can prescribe treatments like prednisolone or hydroxychloroquine to patients who are most likely to benefit. While many of these therapies still require more robust clinical trial data to gain universal acceptance, the move toward treating the underlying immunological environment offers new hope for improving live birth rates for women experiencing the physical and psychological burden of recurrent loss.

## References

[1] Weng J, Couture C, Girard S (2023). Innate and adaptive immune systems in physiological and pathological pregnancy. Biology (Basel).

[2] Zhang Y, Liu Z, Sun H (2023). Corrigendum: fetal-maternal interactions during pregnancy: a 'three-in-one' perspective. Front Immunol.

[3] Mealy G, Brennan K, Killeen SL (2024). Impact of previous pregnancy and BMI on cellular and serum immune activity from early to late pregnancy. Sci Rep.

[4] Raghupathy R, Kalinka J (2008). Cytokine imbalance in pregnancy complications and its modulation. Front Biosci.

[5] Mandal M, Donnelly R, Elkabes S, Zhang P, Davini D, David BT, Ponzio NM (2013). Maternal immune stimulation during pregnancy shapes the immunological phenotype of offspring. Brain Behav Immun.

[6] Saito S (2024). Role of immune cells in the establishment of implantation and maintenance of pregnancy and immunomodulatory therapies for patients with repeated implantation failure and recurrent pregnancy loss. Reprod Med Biol.

[7] Dhar R, Singh S, Sahoo OS (2025). The placental battlefield: viral strategies and immune countermeasures. Front Immunol.

[8] Yosri M, Dokhan M, Aboagye E, Al Moussawy M, Abdelsamed HA (2024). Mechanisms governing bystander activation of T cells. Front Immunol.

[9] Bonney EA (2016). Immune regulation in pregnancy: a matter of perspective?. Obstet Gynecol Clin North Am.

[10] Whiteside SK, Snook JP, Williams MA, Weis JJ (2018). Bystander T cells: a balancing act of friends and foes. Trends Immunol.

[11] Holmgren AM, McConkey CA, Shin S (2017). Outrunning the Red Queen: bystander activation as a means of outpacing innate immune subversion by intracellular pathogens. Cell Mol Immunol.

[12] Arora N, Sadovsky Y, Dermody TS, Coyne CB (2017). Microbial vertical transmission during human pregnancy. Cell Host Microbe.

[13] Saini V, Arora S, Yadav A, Bhattacharjee J (2011). Cytokines in recurrent pregnancy loss. Clin Chim Acta.

[14] Mor G, Cardenas I (2010). The immune system in pregnancy: a unique complexity. Am J Reprod Immunol.

[15] Aghaeepour N, Ganio EA, Mcilwain D (2017). An immune clock of human pregnancy. Sci Immunol.

[16] Hall EJ (2003). The bystander effect. Health Phys.

[17] Flores Ventura E, Esteban-Torres M, Gueimonde M (2025). Mother-to-infant vertical transmission in early life: a systematic review and proportional meta-analysis of Bifidobacterium strain transmissibility. NPJ Biofilms Microbiomes.

[18] Moustakli E, Potiris A, Zikopoulos A (2025). Immunological factors in recurrent pregnancy loss: mechanisms, controversies, and emerging therapies. Biology (Basel).

[19] Guan D, Sun W, Gao M, Chen Z, Ma X (2024). Immunologic insights in recurrent spontaneous abortion: molecular mechanisms and therapeutic interventions. Biomed Pharmacother.

[20] Cai R, Yang Q, Liao Y, Qin L, Han J, Gao R (2025). Immune treatment strategies in unexplained recurrent pregnancy loss. Am J Reprod Immunol.

[21] Practice Committee of the American Society for Reproductive Medicine (2012). Evaluation and treatment of recurrent pregnancy loss: a committee opinion. Fertil Steril.

[22] Odendaal J, Quenby S, Sammaritano L, Macklon N, Branch DW, Rosenwaks Z (2019). Immunologic and rheumatologic causes and treatment of recurrent pregnancy loss: what is the evidence?. Fertil Steril.

[23] Grauerholz KR, Berry SN, Capuano RM, Early JM (2021). Uncovering prolonged grief reactions subsequent to a reproductive loss: implications for the primary care provider. Front Psychol.

[24] Hussain T, Murtaza G, Kalhoro DH (2022). Understanding the immune system in fetal protection and maternal infections during pregnancy. J Immunol Res.

[25] Robertson SA, Care AS, Moldenhauer LM (2018). Regulatory T cells in embryo implantation and the immune response to pregnancy. J Clin Invest.

[26] Shim CH, Cho S, Shin YM, Choi JM (2022). Emerging role of bystander T cell activation in autoimmune diseases. BMB Rep.

[27] Martin MD, Jensen IJ, Ishizuka AS (2019). Bystander responses impact accurate detection of murine and human antigen-specific CD8 T cells. J Clin Invest.

[28] Ianiro G, Niro A, Rosa L, Valenti P, Musci G, Cutone A (2023). To boost or to reset: the role of lactoferrin in energy metabolism. Int J Mol Sci.

[29] Shehata H, Ali A, Silva-Edge M (2022). Thrombophilia screening in women with recurrent first trimester miscarriage: is it time to stop testing? - a cohort study and systematic review of the literature. BMJ Open.

[30] Ali A, Elfituri A, Doumouchtsis SK (2024). Managing couples with recurrent miscarriage: a narrative review and practice recommendations. Int J Gynaecol Obstet.

[31] Shehata H, Elfituri A, Doumouchtsis SK (2023). FIGO Good Practice Recommendations on the use of progesterone in the management of recurrent first-trimester miscarriage. Int J Gynaecol Obstet.

[32] Lakhno IV, Reyes-Lagos JJ, Adam I, Brownfoot FC (2024). Editorial: The repercussions of maternal inflammation in pre-eclampsia on fetal health and neurodevelopment. Front Immunol.

[33] Uzel K, Lakhno I, Turkler C (2023). Tocilizumab is effective in preventing ovarian injury induced by ischemia- reperfusion in rats. An Acad Bras Cienc.

[34] Cavalcante MB, Harrity C, Luu T (2025). 2025 American Society for Reproductive Immunology guidelines for the treatment of recurrent pregnancy losses: practice recommendations from the ASRI Clinical Reproductive Immunology Fellowship. Am J Reprod Immunol.

[35] Rasmark Roepke E, Bruno V, Nedstrand E (2019). Low-molecular-weight-heparin increases Th1- and Th17-associated chemokine levels during pregnancy in women with unexplained recurrent pregnancy loss: a randomised controlled trial. Sci Rep.

[36] Jones AJ, Gokhale PJ, Allison TF (2015). Evidence for bystander signalling between human trophoblast cells and human embryonic stem cells. Sci Rep.

[37] Grafals M, Thurman JM (2019). The role of complement in organ transplantation. Front Immunol.

[38] Priyadarshinee L, Meetei LT, Roshan Singh L (2023). A cross sectional study of pregnancy outcome in women with recurrent pregnancy loss. Int J Reprod Contracept Obstet Gynecol.

[39] Richardson N, Wraith DC (2021). Advancement of antigen-specific immunotherapy: knowledge transfer between allergy and autoimmunity. Immunother Adv.

[40] Lee SK, Kim JY, Lee M, Gilman-Sachs A, Kwak-Kim J (2012). Th17 and regulatory T cells in women with recurrent pregnancy loss. Am J Reprod Immunol.

[41] Danilova N (2006). The evolution of immune mechanisms. J Exp Zool B Mol Dev Evol.

